# An Accessory to the ‘Trinity’: SR-As Are Essential Pathogen Sensors of Extracellular dsRNA, Mediating Entry and Leading to Subsequent Type I IFN Responses

**DOI:** 10.1371/journal.ppat.1000829

**Published:** 2010-03-26

**Authors:** Stephanie J. DeWitte-Orr, Susan E. Collins, Carla M. T. Bauer, Dawn M. Bowdish, Karen L. Mossman

**Affiliations:** Department of Pathology and Molecular Medicine, McMaster University, Hamilton, Ontario, Canada; University of Southern California School of Medicine, United States of America

## Abstract

Extracellular RNA is becoming increasingly recognized as a signaling molecule. Virally derived double stranded (ds)RNA released into the extracellular space during virus induced cell lysis acts as a powerful inducer of classical type I interferon (IFN) responses; however, the receptor that mediates this response has not been identified. Class A scavenger receptors (SR-As) are likely candidates due to their cell surface expression and ability to bind nucleic acids. In this study, we investigated a possible role for SR-As in mediating type I IFN responses induced by extracellular dsRNA in fibroblasts, a predominant producer of IFNβ. Fibroblasts were found to express functional SR-As, even SR-A species thought to be macrophage specific. SR-A specific competitive ligands significantly blocked extracellular dsRNA binding, entry and subsequent interferon stimulated gene (ISG) induction. Candidate SR-As were systematically investigated using RNAi and the most dramatic inhibition in responses was observed when all candidate SR-As were knocked down in unison. Partial inhibition of dsRNA induced antiviral responses was observed *in vivo* in SR-AI/II^-/-^ mice compared with WT controls. The role of SR-As in mediating extracellular dsRNA entry and subsequent induced antiviral responses was observed in both murine and human fibroblasts. SR-As appear to function as ‘carriers’, facilitating dsRNA entry and delivery to the established dsRNA sensing receptors, specifically TLR3, RIGI and MDA-5. Identifying SR-As as gatekeepers of the cell, mediating innate antiviral responses, represents a novel function for this receptor family and provides insight into how cells recognize danger signals associated with lytic virus infections. Furthermore, the implications of a cell surface receptor capable of recognizing extracellular RNA may exceed beyond viral immunity to mediating other important innate immune functions.

## Author Summary

Nearly all viruses produce dsRNA during their replication cycle. This molecule is not normally found in a healthy host cell and thus functions as a flag, alerting the host to a viral infection. Cells can die by lysis during virus infections, and the intracellular dsRNA is then released into the extracellular space. This dsRNA is stable in the extracellular milieu, and is able to function as a signaling molecule, detected by neighboring cells. This has been observed experimentally, as extracellular dsRNA has been used for years to trigger host antiviral responses. It has also been suggested that extracellular dsRNA plays a role in causing pathological symptoms in virus infected patients. Our data suggests that class A scavenger receptors (SR-As) function as cell surface receptors for dsRNA. SR-As bind extracellular, viral dsRNA and mediate its entry into the cell, where it delivers the dsRNA to other known intracellular dsRNA sensors, activating intracellular antiviral responses. These findings shed new light on how the host detects and responds to virus infection.

## Introduction

There is a ‘trinity’ of pattern recognition receptors (PRRs) used by the innate immune system to sense pathogens. These include the toll-like receptors (TLRs), retinoic acid-inducible gene-I (RIG-I)-like receptors (RLRs) and nucleotide oligomerization domain (NOD)-like receptors (NLRs) [1]. All three sensor families have been implicated in innate antiviral responses, with members of each family being able to recognize viral double-stranded (ds) RNA, a pathogen associated molecular pattern (PAMP) and a powerful inducer of both innate and adaptive immune responses. Cellular localization of these dsRNA sensors differs; TLR3 is endosomal while the RLRs and NLRs (RIG-I/MDA-5/LGP2 and Nalp3 respectively) are cytoplasmic [2]–[4]. When dsRNA is endosomal, TLR3 is recruited from the endoplasmic reticulum to the endosome where it binds dsRNA and triggers intracellular signaling pathways through a TRIF dependent mechanism [5]. RIG-I, MDA-5 and LGP2 recognize dsRNA in the cytoplasm and while LPG2 lacks signaling capability [6], RIG-I and MDA-5 signal through interferon (IFN)-β promoter stimulator 1 (IPS-1), an adaptor molecule associated with the mitochondria [7]. These pathways lead to the activation of transcription factors, including IFN regulatory factor (IRF)-3 and -7, and the induction of type I IFNs, IFN stimulated genes (ISGs) and the establishment of an antiviral state [8]. Nalp3 has been shown to mediate dsRNA induced IL-1β production in macrophages, in a TLR3 independent manner, and may play an important role in pro-inflammatory responses to dsRNA [4].

Almost all viruses produce dsRNA sometime during viral replication as either a genomic fragment, a replicative intermediate or by stem and loop structures [9]. Intracellular dsRNA is generated in the virally infected cell. With resulting cell lysis this viral dsRNA is released into the extracellular space, and being a stable (nuclease-resistant) molecule it is able to stimulate antiviral responses in neighboring, uninfected cells [10]. Extracellular dsRNA has been implicated in both local and systemic toxic reactions associated with viral infections, and is an important modulator of both innate and adaptive antiviral immune responses [10]–[12]. In the laboratory the effects of extracellular dsRNA have been observed for many years, as exogenous synthetic dsRNA is commonly used to experimentally induce antiviral responses. Based on cellular localization, RLRs and NLRs are most likely to sense intracellular dsRNA generated during a primary viral infection. TLR3 is expressed on the cell surface of some cell types [13]; however, it is only able to bind dsRNA in low pH environments, such as acidified endosomes [14]. With the existing ‘trinity’ unable to sense extracellular ligands from the cell surface, the outstanding question remains as to how extracellular dsRNA triggers intracellular antiviral responses.

The characteristics of class A scavenger receptors (SR-As) make them attractive candidates for a dsRNA surface receptor to mediate innate antiviral immune responses. Firstly, they are located on the cell surface [15] and can bind nucleic acids [16]. Macrophage SR-As can bind ssRNA molecules, such as poly I and poly G as well as poly IC, a synthetic dsRNA [17]. Secondly, their role in innate immunity is well established, as SR-As are important sensors of bacterial PAMPs. Macrophage expressed SR-As can bind lipopolysaccharide (LPS) and lipoteichoic acid (LTA) molecules associated with gram negative and gram positive bacteria, respectively [18], as well as mediate uptake of bacteria by phagocytosis [19]. Thirdly, SR-As mediate internalization of ligands and subsequent stimulation of intracellular pathways. SR-As mediate uptake of antigens for MHC presentation in T cells [20] and dsRNA molecules targeted for RNAi pathways [21]. Also, exogenous poly IC can induce TNF-α release in RAW 264.7 cells mediated by SR-As [22], and more recently, SR-As were shown to mediate poly IC binding, entry, and induction of a pro-inflammatory response in human epithelial cells [23]. Finally, upon identification of novel SR-A family members, SR-A expression is not restricted to macrophages, as once thought. To date five different species of SR-As have been identified: SR-AI/II/III, MARCO, SCARA3, SCARA4 and SCARA5. Human SR-AI, -AII and –AIII are coded by a single gene and alternative RNA splicing generates the three isoforms [16]. SR-AI/II/III and MARCO are largely expressed on macrophages, but SR-AI/II/III can be detected on endothelial and smooth muscle tissues and MARCO on splenic dendritic cells [16]. SCARA3 is expressed on normal human fibroblasts [24], and SCARA4 and SCARA5 are expressed on endothelial and epithelial cells respectively [25],[26]. With such a broad expression profile, there is likely to be a functional SR-A at the site of most viral infections.

Cell lysis occurs frequently during the course of a viral infection, resulting in the release of the contents of the cell, including intracellularly generated dsRNA, into the extracellular space. Although scavenger receptors have been shown to bind dsRNA, there have been no associations between SR-As and the induction of an antiviral response. This study shows that fibroblasts, non-professional innate immune cells and important IFNβ producers, express functional SR-As. These SR-As act as cell surface receptors for extracellular dsRNA, mediating dsRNA binding and entry, ultimately leading to an antiviral state. An SR-A mediated antiviral response was observed not only *in vitro* in both mouse and human fibroblasts but in an *in vivo* murine model as well. Once internalized by SR-As, extracellular dsRNA elicits antiviral responses through well-characterized dsRNA receptors, namely TLR3, RIG-I and MDA5. Clearly, SR-As play an important role in monitoring the extracellular milieu, and should be considered an essential accessory to TLRs, RLRs, and NLRs for their ability to recognize extracellular dsRNA and mediate its entry and subsequent delivery to intracellular sensors.

## Results

### Murine embryonic fibroblasts express functional SR-As

**Figure 1:**
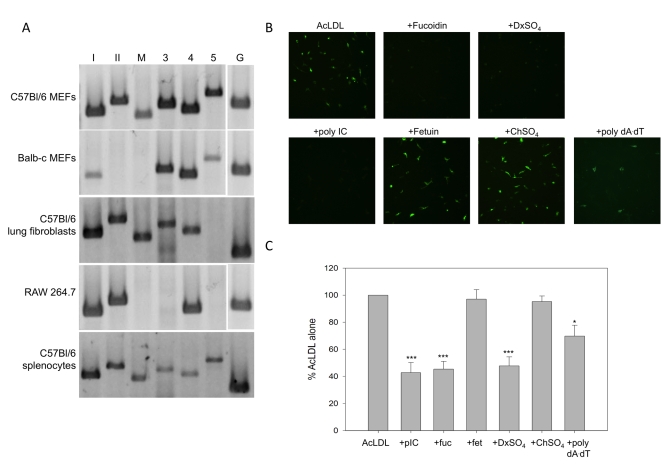
Primary murine fibroblasts express functional SR-As. (**A**) SR-A transcripts were detected using RT-PCR and primers specific to SR-AI (*lane I*), SR-AII (*II*), MARCO (*M*), SCARA3 (*3*), SCARA4 (*4*), and SCARA5 (*5*) in murine embryonic fibroblasts (MEFs) derived from C57Bl/6 or balb-c mice, the macrophage-like cell line RAW 264.7, and primary lung fibroblasts and splenocytes derived from C57Bl/6 mice. GAPDH was used as an internal control for all cell types (*G*). (**B**) Cell associated AcLDL was observed by live-cell fluorescence microscopy in MEFs derived from C57Bl/6 mice treated with Alexafluor 488 labeled AcLDL for 1h (2.5 µg/mL), in the presence or absence of SR-A competitive ligands (fucoidin, DxSO_4_), their corresponding non-competitive counterparts (fetuin, ChSO_4_) or nucleic acids (poly IC, poly dA:dT) all at 100 µg/mL. Magnification 200X. (**C**) AcLDL entry was measured using a fluorescence plate reader assay. Cells were treated with Alexafluor 488 labeled AcLDL (2.5**µg/ mL) for 1h in the presence of poly IC (pIC), fucoidin (fuc), fetuin (fet), DxSO_4_, ChSO_4_ or poly dA:dT (100 µg/mL). Cells treated with AcLDL alone were set at 100% fluorescence. These data include three independent experiments ± SEM. Statistical analysis was performed by a one-way ANOVA with Tukey post test (* p<0.05, *** p<0.001).

Fibroblasts, a non-hematopoietic cell type and key source of IFNβ in response to dsRNA [27], are a relevant cell type in which to study antiviral responses. Therefore the expression profile of SR-As was determined in primary murine fibroblasts. In the absence of available antibodies for all SR-As, the expression profile was generated using RT PCR and gene specific primers (Table 1). The list of candidate SR-As was compiled based on their ability to bind polyanionic ligands. These SR-As include: SR-AI, SR-AII, MARCO, SCARA3, SCARA4 and SCARA5. SRA-III is not expressed on the plasma membrane and is unable to bind polyanionic ligands [28]; therefore it was not pursued as a candidate dsRNA receptor. Despite an initial report suggesting that SCARA3 localization is intracellular, it was included as a candidate SR-A given its ability to bind polyanionic ligands [24]. All six SR-A transcripts tested could be readily detected in C57Bl/6 murine embryonic fibroblasts (MEFs), while SR-A transcript expression in balb-c MEFs was more restricted (Figure 1A). Primary murine lung fibroblasts were found to express all candidate SR-As with the exception of SCARA5. RAW 264.7, a murine monocyte/macrophage cell line, was found to express SR-AI and –AII but not MARCO, as previously described [29],[30]. To our knowledge, expression of SCARA4 transcript is a novel finding for this cell line. Primary murine splenocytes showed levels of expression of all SR-A transcripts. This population of cells would contain those of myeloid lineage, acting as a positive control for SR-A expression. These data suggest that the expression of SR-As at the transcript level is more ubiquitous than previously appreciated.

**Table 1:** Primer sequences used for amplifying scavenger receptor transcripts by RT-PCR.

Species	Gene	Accession number	Sense Primer (5′→ 3′)	Antisense Primer (5′→ 3′)	PCR product length (bp)
Murine	SR-AI	NM_031195	AGAAGAACAAGCGCACGTGG	CCCAACAGCACCCAGGGTTA	578
	SR-AII	NM_001113326	GACACGGAACGCTTCCAGAA	AGCCCGTATATCCCAGCGAT	750
	MARCO	NM_010766.2	CTCCGTCAGCAGTTCAACAA	TCCAGGTTTTCCTTGGTCAC	516
	SCARA3	NM_172604	TGCTATGATGTCAAGGCTGC	GATCATGGAGAGGTTTCGGA	666
	SCARA4	AB038519	CAGCAGGTTTTCCTTCAAGC	TTCTTGATGAGCTGACCGTG	578
	SCARA5	NM_028903	CTGAGACCAGGTTCCCATGT	AAAAATACCGCCGTTAGCCT	865
Human	SR-AI	NM_138715	GACATGGAAGCCAACCTCAT	CCAAGCTCCTACAGACGACC	787
	SR-AII	NM_002445	TCGAGGACTCCCAGGATATG	GGCAGAGAACTGAGGACTGG	446
	MARCO	NM_006770	CAACAAGCTGCTTTTCACCA	ACATCCCTGGGTTCTGAGTG	382
	SCARA3 variant 1	NM_016240	ACGAGATTGAAATTGGCACC	CCCTCATTGGAATCAGAGGA	877
	SCARA3 variant 2	NM_182826	TGCAGCTGGATAACATCTCG	CTTGGTCATCCTGGGCTTTA	510
	SCARA4	NM_130386	CTGCGGACGCTGACCAGCAA	GTGAGGCGGGCAGCCATTGT	762
	SCARA5	NM_173833	CTCTTGAACATGTGCTCCGA	TCACTTGACGTTGCCTCTTG	315

Expression of functional SR-As was determined by investigating acetylated low- density lipoprotein (AcLDL) binding. AcLDL is a well-characterized ligand for SR-AI, –AII and MARCO [16] but not SCARA4 [25] or SCARA5 [26]. To our knowledge, SCARA3′s ability to bind AcLDL has yet to be elucidated. MEFs derived from C57Bl/6 mice were able to bind and take up fluorescently labeled AcLDL (Figure 1B). MEFs derived from balb-c MEFs could also take up AcLDL, but with a lower efficiency (data not shown). The binding of AcLDL was blocked by treatment with SR-A specific competitive ligands, fucoidin and dextran sulfate (DxSO_4_), but not their non-competitive counterparts, fetuin and chondroitin sulfate (ChSO_4_). Interestingly, poly IC, a synthetic dsRNA molecule, was able to inhibit AcLDL binding to a level of inhibition similar to the classic SR-A ligands. DsRNA mediated inhibition was not limited to poly IC, as *in vitro* transcribed dsRNA of different lengths inhibited AcLDL binding with similar efficiency (data not shown). A dsDNA molecule, poly dA:dT could partially inhibit AcLDL binding. AcLDL entry, as measured by a fluorescence plate reader assay, was inhibited by poly IC, fucoidin and DxSO_4_, but not fetuin or ChSO_4_, in a statistically significant manner (Figure 1C). Inhibition by poly dA:dT was statistically significant but clearly not as effective as dsRNA and the classic SR-A ligands.

### SR-As mediate dsRNA binding, entry and subsequent ISG expression

**Figure 2:**
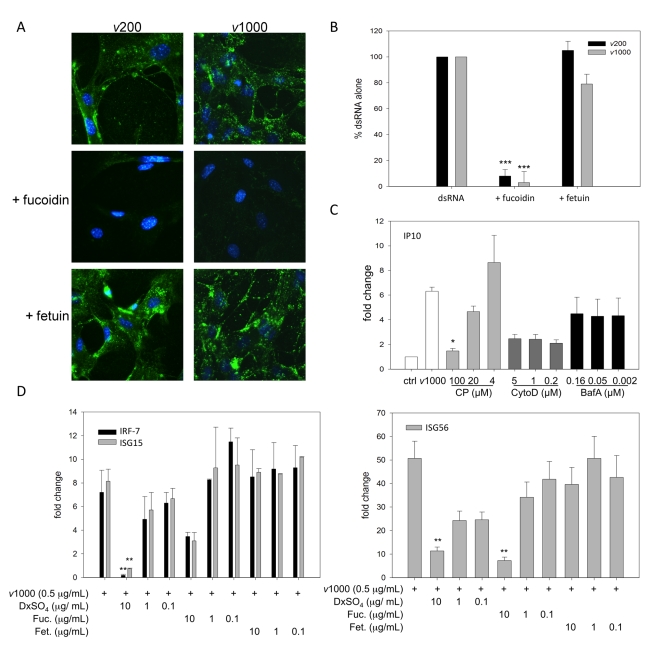
SR-As mediate viral dsRNA binding, entry and resulting ISG induction in MEFs using a mechanism dependent on clathrin-mediated endocytosis. (**A**) Cellular binding of viral dsRNA was observed by fluorescence microscopy in MEFs derived from C57Bl/6 mice treated with either 200 bp or 1000 bp Alexafluor 488 labeled viral dsRNA (*v*200 or *v*1000 respectively, both at 1 µg/mL) for 1h, in the presence or absence of fucoidin or fetuin (both at 100 µg/mL). Cells were fixed and nuclei counterstained with Hoechst 33258. Magnification 400X. (**B**) Fluorescently labeled dsRNA entry was quantified using cells treated with Alexafluor 488 labeled *v*200 or *v*1000 (both at 1 µg/mL) for 1h in the presence of fucoidin or fetuin (both at 100 µg/mL). Cells treated with dsRNA alone (dsRNA) were set at 100% fluorescence. These data include three independent experiments ± SEM. Statistical analysis was performed by a one-way ANOVA with Tukey post test (*** p<0.001) (**C**) MEFs were treated with *v*1000 (1 µg/mL) for 2h in the presence of endocytosis pathway inhibitors, chlorpromazine (CP), cytochalasin D (cyto D) and bafilomycin A1 (Baf A). IP10 transcript levels were measured using real time PCR and reported as a fold change difference from mock treated cells (ctrl), whose fold change was set to 1. These data are the average of three independent experiments ± SEM. Statistical analysis was performed by one-way ANOVA with a Dunnett's post test using *v*1000 as the control comparison, * p<0.05 (**D**) ISG15, IRF-7 and ISG56 transcript levels were measured by real time PCR in C57Bl/6 MEFs treated with viral dsRNA (*v*1000, 0.5 µg/mL) for 4 h in the presence or absence of dextran sulfate (DxSO_4_), fucoidin (Fuc.) or fetuin (Fet.). These data are the average of three independent experiments ± SEM. Statistical analysis was performed by one-way ANOVA and a Dunnett's post test, comparing all treatments to *v*1000 alone, ** p<0.01.

As dsRNA was able to block AcLDL binding and uptake, the possibility that SR-As mediate extracellular dsRNA entry was investigated. DsRNA molecules were derived by *in vitro* transcription based on randomly selected West Nile virus genome sequences. Two lengths were used to reveal any differences in binding efficiencies, which had previously been observed with other dsRNA sensors. In the absence of the competitive ligands fluorescently labeled dsRNA was found to associate with the MEFs (Figure 2A). Fucoidin blocked dsRNA cell association, while fetuin did not. Similar results were observed with DxSO_4_ and ChSO_4_ (data not shown). Furthermore, dsRNA entry was quantified using a fluorescence plate reader assay (Figure 2B). Corroborating the observations made by fluorescence microscopy, it was found that fucoidin almost completely blocked dsRNA binding and uptake while at the same concentration fetuin did not. These results were similar between the two dsRNA lengths tested. Balb-c MEFs were also able to bind fluorescently labeled dsRNA (both *v*200 and *v*1000), which was blocked by fucoidin but not fetuin (Figure S1).

The mechanism of dsRNA entry was investigated using pharmacological inhibitors. Cells were pre-treated with chlorpromazine, a clathrin-mediated endocytosis inhibitor [31], cytochalasin D, an actin polymerization inhibitor [32], and bafilomycin A1, a specific V-H-ATPase inhibitor [21], for 30 minutes. Cells were then treated with *v*1000 for 2h in the presence of the inhibitors. Transcript levels of IP10, an early ISG, were used as an indicator of dsRNA effects within the cell (Figure 2C). Chlorpromazine blocked induction of IP10 transcript expression in a concentration dependent manner; while bafilomycin A1 did not alter levels when compared to *v*1000 alone. Although not statistically significant, cytochalasin D had a general suppressive effect that was concentration independent. No changes in cell viability were detected with any of the inhibitors using the fluorescent cell viability dyes alamar Blue and CFDA-AM (data not shown). These data suggest that similar to other ligands, SR-A mediated dsRNA entry occurs by clathrin mediated endocytosis.

The inhibition of dsRNA entry in the presence of SR-A specific competitive ligands corresponded with a decrease in ISG induction as measured by real time RT-PCR (Figure 2D). ISG transcripts were measured in C57Bl/6 MEFs treated with *v*1000 for 4h in the presence or absence of DxSO_4_, fucoidin, or fetuin after a 30 minute pretreatment with the inhibitors alone. DxSO_4_ blocked induction of ISG15, IRF-7 and ISG56 transcripts by *v*1000 in a concentration dependent manner. Though not statistically significant for ISG15 and IRF-7, similar results were observed with fucoidin. DxSO_4_ (10 µg/mL) was able to completely inhibit *v*1000 induced IFN-β induction while fucoidin (100 µg/mL) reduced IFN-β induction but could not block it completely (data not shown). It is this small amount of IFN that is likely responsible for the increased ISG induction observed with *v*1000 and fucoidin. Treatment with fetuin, the corresponding non-competitive compound, did not affect ISG transcript expression. Thus, SR-As appear to be the chief mediator of extracellular dsRNA binding, entry and resulting ISG induction in MEFs.

### SR-As compensate for one another with regards to dsRNA binding

**Figure 3:**
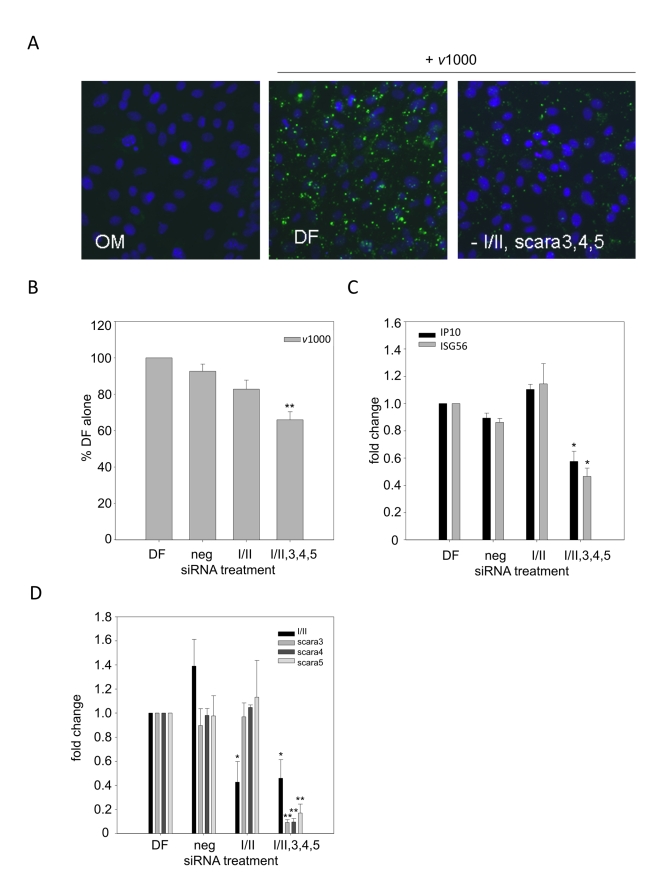
dsRNA binding is dependent on all candidate SR-A family members. DsRNA binding, entry and subsequent induction of ISGs was measured in balb-c derived MEFs treated with media alone (OM), Dharmafect alone (DF), SR-AI/II specific siRNA (I/II) or a combination of SR-AI/II, SCARA3, SCARA4 and SCARA5 siRNA oligomers (-I/II,3,4,5) for 24h. (**A**) Binding of dsRNA was measured in MEFs treated with SR-A siRNA for 24h followed by Alexafluor 488 labeled *v*1000 (1 µg/mL) for 1h. Nuclei were counterstained with Hoechst 33258. Magnification 400X. (**B**) Entry of Alexafluor 488 labeled *v*1000 was measured by fluorescence plate reader, in balb-c MEFs treated with siRNA for 24h followed by 1h treatment with fluorescently labeled *v*1000 (1 µg/mL). Results are reported as a % of DF alone (set to 100%). These data include three independent experiments and are reported as an average ± SEM. Statistical analysis was performed by a one-way ANOVA with a Dunnett's post test, with DF being the control comparison (** p<0.01). (**C**) Levels of ISG56 and IP10 transcript expression were measured by real time PCR in balb-c MEFs treated with SR-A siRNA for 24h followed by stimulation with 1 µg/mL *v*1000 for 6 h. Results are reported as a fold change difference from DF treated cells (DF), whose fold change was set to 1. These data include three independent experiments and are reported as an average ± SEM. Statistical analysis was performed by a one-way ANOVA with a Dunnett's post test, with DF being the control comparison (* p<0.05). (**D**) SR-A transcript levels were measured by real time PCR in balb-c MEFs treated with SR-A siRNA for 24h. Results are reported as a fold change difference from DF treated cells (DF), whose fold change was set to 1. These data include three independent experiments and are reported as an average ± SEM. Statistical analysis was performed by a one-way ANOVA with a tukey's *post hoc* test (* p<0.05, ** p<0.01).

SR-A species were knocked down individually and in combinations by siRNA to determine whether specific SR-As were responsible for binding extracellular dsRNA. MEFs derived from balb-c mice were used as they bound dsRNA similar to C57Bl/6 derived MEFs but do not express SR-AII and MARCO, removing these two SR-As as possible individual candidates. Pre-validated siRNA oligos were used to knock down SR-AI, SCARA3, SCARA4 and SCARA5. The SR-AI oligo sequence and downstream real time PCR primers target the SR-AII species as well; therefore the siRNA knockdowns using this oligo are reported as SR-AI/II. Following knockdown, dsRNA binding, entry and ISG induction were measured. No effects were observed when individual SR-As were knocked down; SR-AI/II results are shown as a representative of results observed by single knockdowns. The most significant effects were observed when all candidate SR-As were knocked down in combination (Figure 3). When SRAI/II, SCARA3, SCARA4 and SCARA5 were knocked down together (Figure 3D) binding of fluorescently labeled dsRNA was blocked considerably as observed by fluorescence microscopy (Figure 3A). The negative control (non-targeting negative control pool) did not affect dsRNA binding (data not shown). A statistically significant decrease in dsRNA entry (Figure 3B) and downstream ISG induction (Figure 3C) was also observed. As an effect was only observed when all relevant SR-As were knocked down, these data suggest that SR-As have a remarkable ability to compensate for one another.

### Antiviral responses are impaired in SR-AI/II knockout mice

**Figure 4:**
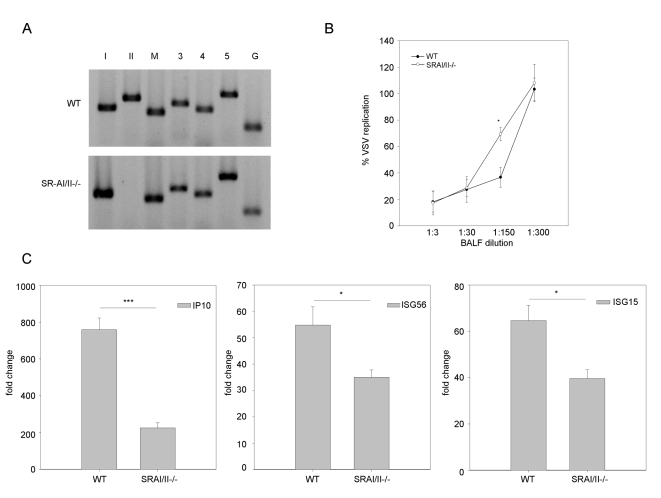
SR-As are involved in dsRNA induced antiviral responses *in vivo*. (**A**) SR-A expression was measured at the transcript level in both wild type (WT) and SR-AI/II^-/-^ mouse whole lungs using RT-PCR and primers specific to SR-AI (*lane I*), SR-AII (*II*), MARCO (*M*), SCARA3 (*3*), SCARA4 (*4*), SCARA5 (*5*) and GAPDH (G). (**B**) Poly IC treated mice were sacrificed 12h post-treatment and type I IFN bioactivity in the BALF was determined by antiviral assay. Data is presented as % VSV replication at the indicated BALF dilutions. The absence of VSV replication indicates the presence of type I IFN in BALF. Statistical analysis was performed using a one-way ANOVA with a tukey's *post hoc* test (* p<0.05). (**C**) IP10, ISG56 and ISG15 transcript levels in the lung of poly IC treated WT and SR-AI/II^-/-^ mice were measured 12h post treatment. Results are reported as a fold change comparison with PBS treated WT or SR-AI/II^-/-^ mice. Statistical analysis was performed using a t test; *** p<0.001, * p<0.05. Data show one representative experiment and are reported as means ± SEM, n = 4.

A role for SR-As in dsRNA-induced antiviral responses was studied *in vivo* using SR-AI/II ^-/-^ mice [33]. By RT-PCR it was determined that all candidate SR-As were present at the transcript level in whole lung tissue from WT mice (Figure 4A). SRA-I/II^-/-^ mice have a disruption at exon 4, which is essential for trimerization of the receptor [33]. The forward primer for SR-AII in our expression panel falls in exon 4 (the reverse in exon 10) while the SR-AI primers are specific for sequences in exons 5 and 10. Thus the SR-AI/II^-/-^ mice showed expression of MARCO, SCARA3, SCARA4 and SCARA5 and disruption of SRAI/II as expected. Both WT and SR-AI/II ^-/-^ mice were treated with PBS or poly IC (50 µg) by intranasal administration. At 12 h post-treatment, type I IFN bioactivity was measured in broncho-alveolar lavage fluid (BALF). Naïve MEFs were treated with BALF diluted in growth media and after 24 h cells were challenged with a VSV-GFP infection. There was a statistically significant difference in antiviral responses between WT and SRAI/II ^-/-^ BALF as determined by serial dilution, suggesting less type I IFN was produced in poly IC treated SRAI/II^-/-^ mice compared with WT mice (Figure 4B). No antiviral activity was detected in BALF from PBS treated mice (data not shown). Poly IC also induced lower levels of ISG transcripts (IP10, ISG56 and ISG15) in knockout mice when compared to WT mice, as determined by real time PCR. Although the most dramatic decrease was observed with IP10, all three genes tested showed statistically significant decreases in transcript levels when compared with WT (Figure 4C). These data show that SR-AI/II plays a role in mediating antiviral responses induced by exogenous dsRNA *in vivo*. This inhibition is partial, which would be expected considering the presence of other SR-As within the lung.

### SR-A mediated antiviral mechanisms involve classic dsRNA PRRs

**Figure 5:**
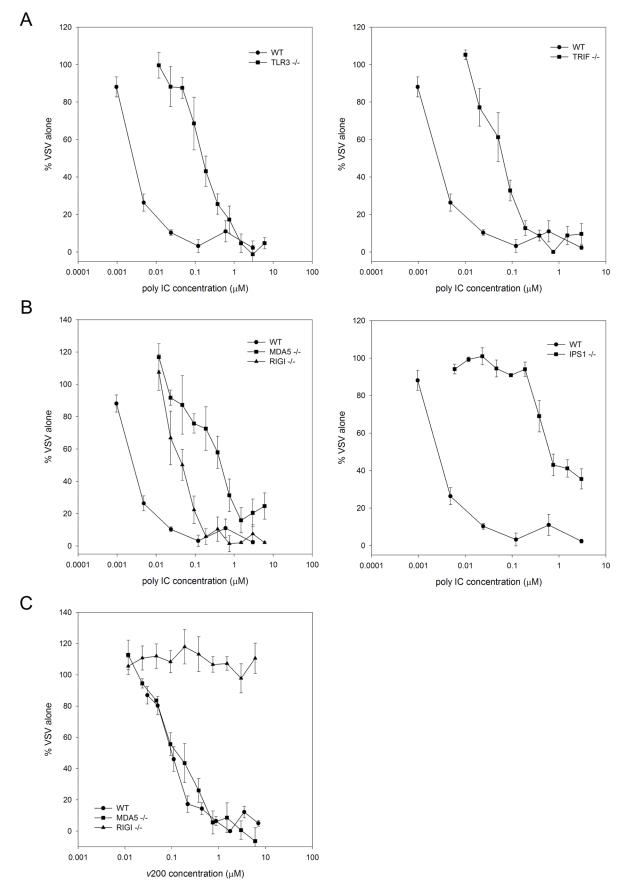
DsRNA induced antiviral responses are mediated by TLR3 and the RLRs. MEFs derived from (**A**) wild type C57Bl/6 (WT), TLR3^-/-^, TRIF^-/-^ and (**B**) WT, RIGI^-/-^, MDA5^-/-^ and IPS-1^-/-^ mice were treated with serially diluted nM concentrations of poly IC for 6 h. (**C**) To compare length effects, WT, RIGI^-/-^ and MDA5^-/-^ MEFs were treated with serially diluted nM concentrations of *in vitro* transcribed dsRNA (*v*200). Cells were then challenged with VSV-GFP (MOI = 0.1) and GFP fluorescence intensity was measured at 24h pi. Data is reported for each dsRNA concentration tested as a % of VSV replication in mock treated cells. These data include three independent experiments and are reported as an average ± SEM.

A functional role for SR-As is to act as a carrier, delivering ligands to downstream pathways [16]. SR-A delivery of extracellular dsRNA to intracellular sensors was investigated in MEFs deficient for the classic intracellular dsRNA PRRs, TLR3, RIG-I and MDA5, as well as their downstream adapter molecules, TRIF or IPS-1. An antiviral assay was performed whereby cells treated with a range of dsRNA concentrations for 6h were infected with VSV-GFP and the resultant fluorescence, representing viral replication, was quantified 24h pi (Figure 5). The antiviral response to extracellular dsRNA was similarly reduced in TLR3 and TRIF null MEFs relative to WT MEFs (Figure 5A). Poly IC induced antiviral responses were significantly reduced in both RIG-I and MDA5 null MEFs, with a greater inhibition observed in MDA5^-/-^ MEFs. Poly IC induced responses were also inhibited in IPS-1^-/-^ MEFs, which corroborates the receptor data, as both RIG-I and MDA5 signal through IPS-1 (Figure 5B). The poly IC used in this study is a mixture of dsRNA molecules with varying lengths, with an average of 4000 bp and a range between 400 bp and >6000 bp (data not shown). As dsRNA recognition is length dependent [2], a dsRNA molecule of a defined length was included to ensure that SR-As delivered extracellular dsRNA to the appropriate intracellular PRR. Antiviral responses induced by *v*200, an *in vitro* transcribed dsRNA molecule 200 bp in length, were completely dependent on RIG-I and independent of MDA5 (Figure 5C). These results suggest that extracellular dsRNA induced antiviral responses are mediated by both TLR3 and the RLRs in a length dependent manner.

### SR-As mediate dsRNA entry in human fibroblasts

**Figure 6:**
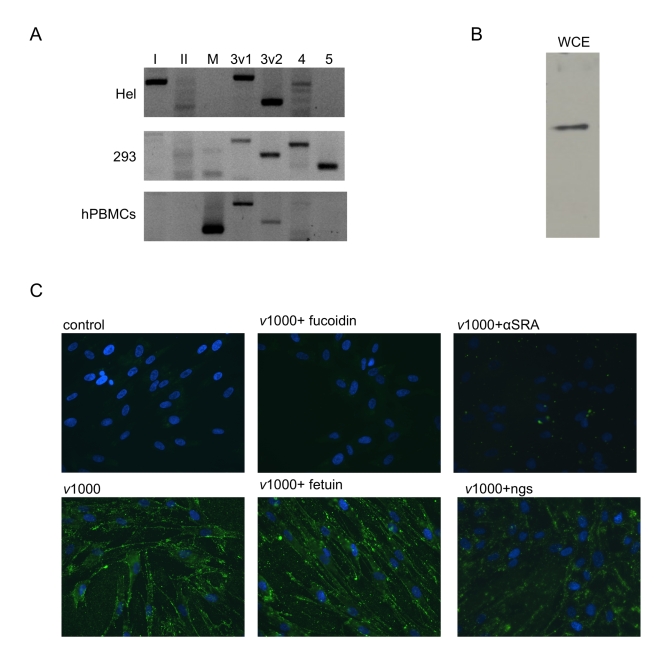
Human fibroblasts express SR-As that mediate dsRNA binding via the collagenous domain. (**A**) SR-A transcripts were detected in primary human fibroblasts (Hel) using RT-PCR and primers specific to SR-AI (*lane I*), SR-AII (*II*), MARCO (*M*), SCARA3 variant 1(*3v1*), SCARA3 variant 2 (3v2), SCARA4 (*4*), and SCARA5 (*5*). SR-A transcripts detected from immortalized human embryonic kidney cells (293) and primary human peripheral blood mononuclear cells (hPBMCs) were included for comparison purposes. (**B**) SR-A expression at the protein level was measured in human fibroblasts by western blot analysis using a whole cell extract (WCE). A single band ∼70kDa in size was detected. (**C**) Alexafluor 488 labelled *v*1000 (1 µg/mL) binding was observed in HEL cells by fluorescence microscopy with or without pre-treatment with the SR-A competitive ligand fucoidin or fetuin, its non-competitive counterpart (both at 100 µg/mL). Also, *v*1000 binding was monitored in the presence of the anti-human SRA antibody (αSRA) and a normal goat serum (ngs) control (both diluted at 1∶50).

To assess whether SR-As mediate dsRNA entry in human fibroblasts, SR-A responses were investigated in a primary human fibroblast cell type (HEL). SR-AI, SCARA3 variant 1 and 2 and SCARA4 could be detected at the transcript level using RT PCR (Figure 6A). For comparison purposes, SR-A transcript levels were also investigated in 293, a human embryonic kidney cell line, and in primary human peripheral blood mononuclear cells (hPBMCs). 293 cells were found to express SCARA3 variant 1 and 2 as well as SCARA4 and SCARA5 at the transcript level, while hPBMCs express MARCO and SCARA3 variant 1 and 2. A cloned hSR-AII sequence acted as a positive control for the SR-AII primers. Further study in HEL cells showed that an SR-A protein (∼70 kDa) could be detected from whole cell extracts using a polyclonal antibody specific to the SR-A collagenous domain (Figure 6B). Binding of fluorescently labeled dsRNA (*v*1000) was observed by fluorescence microscopy. As with murine fibroblasts (Figure 2A), extracellular dsRNA binding was blocked by fucoidin but not fetuin (Figure 6C). Furthermore, the anti-human SR-A polyclonal antibody blocked *v*1000 binding, while the normal goat serum (ngs) control did not. DsRNA-induced IP10 transcript expression was inhibited 75.94±4.71% (n = 3) by the anti-human SR-A antibody compared with the ngs control, as measured by real time PCR (data not shown). It should be noted that human fibroblasts were able to bind AcLDL with moderate efficiency, similar to balb-c MEFs (data not shown). These results suggest that human fibroblasts express SR-As that mediate dsRNA binding and downstream ISG induction, similar to murine fibroblasts.

## Discussion

It is well established that dsRNA is a potent signaling molecule and modulator of innate immune responses. Extracellular dsRNA plays an important role in these functions; however, its mechanism of signaling intracellular antiviral pathways has remained largely unknown. In this study we propose that SR-As function as surface receptors for dsRNA, mediating its entry into the cell and delivery to known intracellular PRRs, resulting in downstream type I IFN and ISG production. There is already a precedent for SR-As binding dsRNA. In the 1980s it was found tfhat SR-As expressed by macrophages could bind poly IC [17]. The novelty of this study is threefold. Firstly, this study demonstrates that SR-A expression is broader than previously appreciated. Secondly, the present study shows that ligand binding by SR-As is compensatory. Thirdly, these data show that SR-As are essential mediators of extracellular dsRNA induced antiviral responses. The implications of these data are important for understanding both viral pathogenesis and host responses.

To our knowledge, this study represents the most complete examination of all known class A scavenger receptors within one cell type. MEFs were studied because they are commonly used for investigating antiviral responses *in vitro*, particularly when using knockout mice. Also, fibroblasts are an important producer of IFNβ, which is a key modulator of innate immune responses [27]. In this study we found that transcript levels of almost all candidate SR-As were high in MEFs and primary adult lung fibroblasts, even SR-As such as SR-AI, -AII and MARCO, whose expression was thought to be restricted to macrophages. Furthermore, C57Bl/6 derived MEFs were able to bind AcLDL, a ligand specific to SR-AI/II and MARCO. As expected, AcLDL binding was blocked by the SR-A competitive ligands fucoidin and DxSO_4_, but not the corresponding non-competitive ligands, fetuin and ChSO_4_. Considering the present data, fibroblasts should now be included as an SR-A expressing cell type, therefore extending expression of SR-As to another non-immune cell type, and further expanding their potential influence in innate immune responses.

The ability of poly IC to compete with AcLDL for binding at the cell surface suggested that dsRNA was binding an SR-A. This hypothesis was investigated using fluorescently labeled *in vitro* transcribed dsRNA. Determined by microscopy and a fluorescence plate reader assay, dsRNA of both 200bp and 1000bp lengths (*v*200 and *v*1000) bound to cells. The SR-A competitive ligands fucoidin and DxSO_4_ almost completely displaced fluorescently labeled dsRNA binding and entry in C57Bl/6 MEFs while their non-competitive counterparts, fetuin and ChSO_4_ did not. The competitive ligands also inhibited downstream ISG induction. Similar results were previously observed in human epithelial cells, where poly IC binding and down stream induction of pro-inflammatory cytokines was displaced by the SR-A competitive ligands DxSO_4_ and fucoidin, but not fetuin and heparin (another non-competitive SR-A ligand) [23]. With regards to dsRNA entry, it is known that SR-As enter cells through an endocytic pathway [16], and it has been shown that SR-A mediated gene expression was dependent on endocytosis in macrophages [34]. The pharmacological inhibitor data with chlorpromazine suggests that dsRNA entry appears to be an active process through clathrin-mediated endocytosis. The suppressive quality of cytochalasin D, though not statistically significant, hints at a role for actin in dsRNA uptake. The inability of bafilomycin A1 to completely block dsRNA-mediated ISG induction suggests that SR-A-mediated binding and uptake of dsRNA are independent of endosomal acidification, suggesting a component of extracellular dsRNA mediated ISG induction is TLR3 independent. These entry characteristics are the same as those found with poly IC acting as an SR-A ligand in RAW 264.7 cells. Poly IC induced TNF-α production required internalization by clathrin coated pit formation and actin polymerization, but not endosomal acidification [22]. In a more recent study, dsRNA entry was found to be clathrin mediated and dependent on actin polymerization in dendritic cells [35]. These data suggest that SR-As are the chief surface receptors for extracellular dsRNA and that these receptors are mediating dsRNA entry by clathrin mediated endocytosis resulting in subsequent ISG induction.

As there were multiple candidate SR-A species that could act as a surface receptor for dsRNA, siRNA was used to knock down each species to investigate their role in dsRNA binding. Effects were investigated in balb-c MEFs as they were found to bind dsRNA similarly to C57Bl/6 MEFs but have a more restricted SR-A expression profile, expressing only SR-AI, SCARA3, SCARA4 and SCARA5. No effect was observed when individual SR-As were knocked down and the most significant effects were observed when all SR-As were knocked down together (Figure 3). With significant knock down of all present SR-As there was a statistically significant decrease in dsRNA binding, entry and ISG induction. These data suggest that no individual SR-A is responsible for binding dsRNA, and that all candidates if present can compensate for one another with regards to binding extracellular dsRNA. This conclusion is not unexpected, as all known SR-As, with the exception of SR-AIII, contain a collagenous structure, which is responsible for binding polyanionic ligands such as nucleic aids [28]. From the neutralization studies in human fibroblasts it is likely that SR-As bind extracellular dsRNA via their collagenous domain. Knock down using siRNA was not complete and as the minimal levels of SR-A expression required to mediate binding and uptake of dsRNA is unknown, residual levels of SR-A expression could explain the partial inhibition observed. As new SR-As are continually being discovered (SCARA5 was identified in 2006) it is also likely that other, yet to be identified SR-A members could be present and binding extracellular dsRNA.

SR-As also mediated dsRNA induced antiviral responses *in vivo*. When mice were treated with poly IC by intranasal administration, SRAI/II^-/-^ mice showed less type I IFN production and lower ISG transcript levels when compared with their WT counterparts. This inhibition was partial, which would be expected, as MARCO, SCARA3, SCARA4 and SCARA5 transcripts could all be detected in the lung. Similar results have been previously reported with regards to the inflammatory response; poly IC treated SRAI/II^-/-^ mice demonstrated lower levels of infiltrating polymorphonuclear leukocytes and lower transcript levels of pro-inflammatory cytokines when compared to WT controls [23]. These data show that SR-A mediated antiviral responses to extracellular dsRNA are not limited to fibroblasts but are involved in complex systems as well. It is likely that one cell type is not solely responsible for the inhibitory effect observed *in vivo*. It is possible that SR-As in the lung epithelium, in combination with SR-As on recruited immune cells, could collectively be responsible for sensing extracellular dsRNA and mediating the observed antiviral response.

SR-As can act as carriers, bringing ligands to intracellular signaling pathways [16]. As SR-As appear to have an essential role in dsRNA uptake into the cell via endocytosis, the question arises as to the mechanism of dsRNA signaling once inside the cell. Considering the role of SR-As as a carrier, it has been previously shown that SR-As can cooperate with TLRs. SR-As are able to mediate apoptosis in a TLR4-dependent manner [36] and MARCO can deliver CpG DNA to endosomal TLR9 [37]. The present data suggest that SR-As may be delivering extracellular dsRNA to endosomes to be recognized by TLR3. The antiviral response was not completely inhibited in TLR3^-/-^ and TRIF^-/-^ MEFs, however, suggesting that there is a TLR3/TRIF independent component to the extracellular dsRNA induced antiviral response. RIGI^-/-^, MDA5^-/-^ and IPS1^-/-^ MEFs showed inhibition in antiviral responses compared with WT. As the poly IC preparation used in this study contains mostly larger dsRNA molecules, the antiviral response was more dependent on MDA5 than RIG-I. As expected, the antiviral response to a short dsRNA molecule of defined length was dependent on RIG-I but not MDA-5. There was even greater dependence on IPS-1, the adapter molecule utilized by both the RLRs. These data demonstrate that extracellular dsRNA signals through cytoplasmic dsRNA sensors. These data are similar to data reported *in vivo* with poly IC treated IPS-1^-/-^ and TRIF^-/-^ mice. The extracellular dsRNA (poly IC injected i.p. without a lipid based transfection reagent) induced type I IFNs and ISGs (IP10), which were partially inhibited in TRIF^-/-^ mice but completely abrogated in IPS-1^-/-^ mice [38].

**Figure 7:**
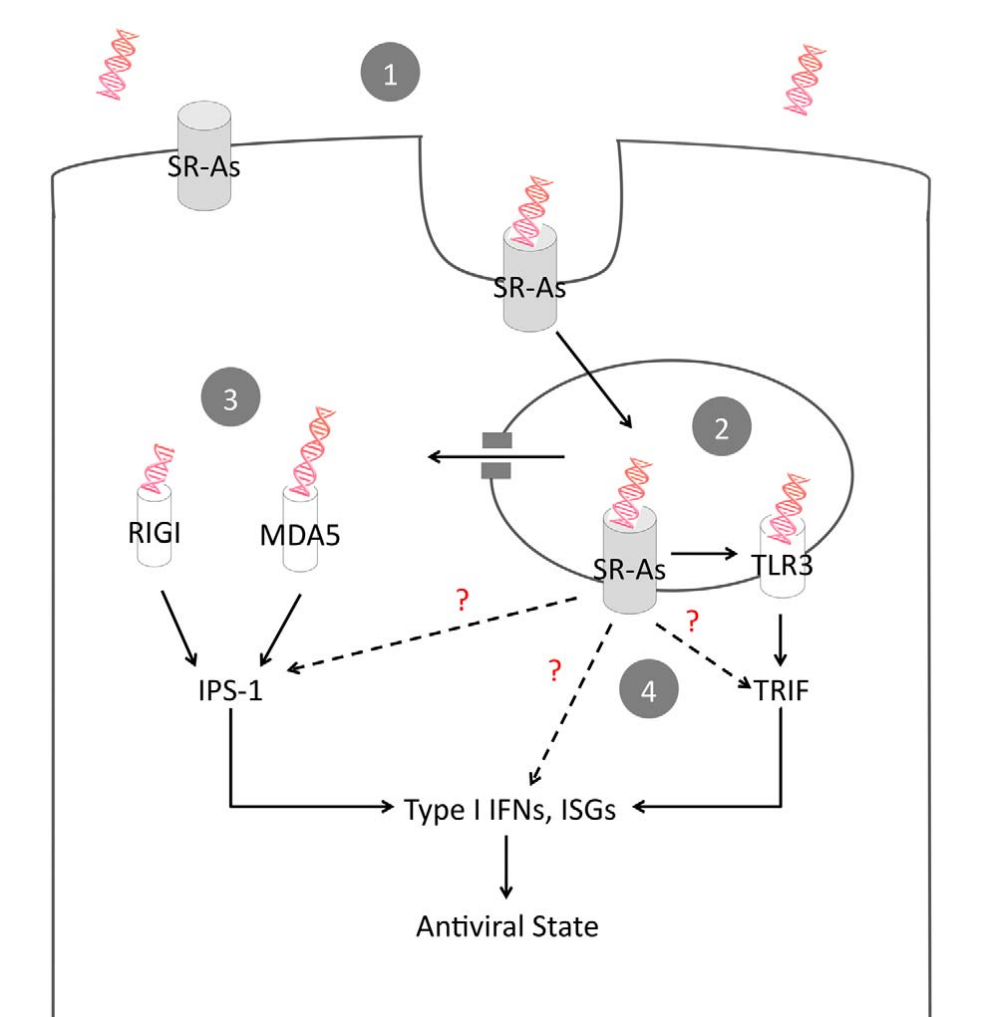
Proposed model of SR-A-mediated antiviral activity. Lytic viral infections lead to dsRNA in the extracellular environment. (**1**) Surface expressed SR-As bind extracellular dsRNA and facilitate entry via clathrin-mediated endocytosis. (**2**) Endosomal TLR3 binds dsRNA and induces an antiviral response via TRIF. (**3**) dsRNA escapes the endosome and is detected in the cytoplasm by the RLRs RIG-I and MDA5, which induce an antiviral response via IPS-1. (**4**) While the carrier function of SR-As is likely the dominant mode of action, the direct contribution of SR-As to antiviral immunity by binding to TRIF and IPS-1 or by inducing independent, non-classical antiviral responses cannot be ruled out.

Therefore, we propose a mechanism of action whereby surface expressed SR-As mediate the entry of extracellular dsRNA via clathrin-mediated endocytosis (Figure 7). Once within the endosome, dsRNA can be detected by TLR3, which would activate a TRIF-dependent antiviral response. In addition, the cytoplasmic sensors RIG-I and MDA-5 play an important role in mediating antiviral responses to extracellular dsRNA. It is currently unclear, however, how dsRNA within the endosome becomes available to cytosolic sensors. While we cannot formally exclude the possibility that SR-As directly interact with the adaptor molecules TRIF and IPS-1, our data suggest that SR-As predominantly function as carriers to deliver dsRNA to TLR3, RIG-I and MDA-5. Furthermore, there is a possibility that SR-As are capable of influencing the antiviral response independent of TLRs and RLRs. We recently found that an antiviral state can be established with high poly IC concentrations independent of IPS-1 and the downstream transcription factor IRF3 [39]. SR-A ligand binding causes tyrosine phosphorylation of phospholipase C-γ1 (PLC-γ1) and phosphatidylinositol 3-kinase (PI 3-kinase) activation, as well as activation of pathways involving protein kinase C (PKC) and mitogen-activated protein kinases (MAPKs) [16]. The mechanisms behind these signaling pathways and the link between endosomal dsRNA and cytosolic sensors are currently under investigation.

The data in this study demonstrate an essential role for SR-As in mediating dsRNA entry, but the downstream signaling capabilities of the SR-As remain unclear. It is possible that instead of being limited to functioning as a delivery system, SR-As could function as pattern recognition receptors in their own right, modulating antiviral responses similar to the trinity of PRRs. A legitimate pattern recognition receptor can discriminate between self and non-self, with high specificity. The trinity of dsRNA PRRs does this through specific cellular localization and ligand binding specificities. It could be argued that SR-As would be able to discern self vs. non-self based on cellular localization as well. Being surface receptors, SR-As sense extracellular nucleic acids, which would only be extracellular during non-homeostatic situations, such as pathogen-mediated cell lysis. With regards to specificity, a study investigating SR-A ligand characteristics showed that SR-As do not bind ligands based solely on their charge [17]. This study reports that multiple negative charges are a necessary but not sufficient requirement for SR-A binding [17]. In fact, within RNA species, some polypurines demonstrate high affinity for SR-AI/II (polyinosinic acid, polyguanylic acid) while other polypurines do not (polyadenylic acid). In the present study it was found that a negatively charged nucleic acid, poly dA:dT (dsDNA) only partially inhibited AcLDL binding. These data show a preference for specific polyanionic nucleic acids, a specificity that is poorly understood. Another characteristic of a dsRNA PRR would be its conservation between species, and preliminary evidence suggests that SR-As mediate extracellular uptake and downstream ISG induction in both human and murine fibroblasts. Although hypothetical at this point, the possibility that SR-As function as a dsRNA PRR modulating antiviral responses independent of TLRs, RLRs and NLRs is intriguing and is currently under investigation.

A surface receptor for dsRNA also changes the perspective on extracellular dsRNA. The idea that RNA can act as an extracellular signaling molecule opens many possibilities not only for antiviral responses but also other aspects of innate immunity. DsRNA is a remarkably stable nucleic acid, able to withstand host nuclease activity [10]. With the existence of an extracellular dsRNA receptor, it is possible that dsRNA could act in a paracrine fashion to induce an antiviral state in neighboring cells. RNA as a ‘danger signal’ may in fact not be limited to viral infections. Tens of thousands of long non-coding (nc)RNAs, RNAs longer than 200 nucleotides, are present in mammalian cells [40]. If a cell dies in an uncontrolled manner, whether it is virus induced or by another stimulus, the contents of the cell, including these ncRNAs, would be introduced into the extracellular space and subsequently detected by neighbouring cells through their SR-As. Recently evidence for this hypothesis has been found in mice, where necrotic cell death stimulated TLR3 activation and subsequent inflammation, independent of viral infection [41]. As an alternative to RNA ligands, recently it has been shown that the dsRNA binding receptor in dendritic cells can also bind CpG ODNs, suggesting that the SR-As could mediate recognition of this important bacterial PAMP [35]. These ideas require further investigation but suggest that SR-As could play a part in innate immunity that extends far beyond the present antiviral observations.

## Materials and Methods

### Ethics statement

All animals were handled in strict accordance with good animal practice as defined by the Canadian Council for Animal Care, and all animal work was approved by the McMaster Animal Research Ethics Board.

### Cells and materials

Polyinosinic/polycytidylic acid (poly IC) was purchased from GE Healthcare (Buckinghamshire, UK). Chlorpromazine hydrochloride, cytochalasin D, bafilomycin A1, dextran sulfate, chondroitin sulfate, fucoidin, fetuin, and polydeoxyadenylic acid^.^polythymidylic acid (dA:dT) were purchased from Sigma (Oakville, Canada). Real-time PCR Taqman probes for murine ISG15, IRF-7, ISG56, IFNβ, IP10, SR-AI/II, SCARA3-5, GAPDH and human IP10 and GAPDH were purchased from Applied Biosystems (Streetsville, Canada). SiRNA oligomers against SR-AI/II, SCARA3-5, a non-targeting negative control pool, and the Dharmafect transfection reagent were all purchased from Dharmacon (Lafayette, CO). The anti-human SR-A antibody was purchased from Millipore (Billerica, MA). Vesicular stomatitis virus expressing green fluorescent protein (VSV-GFP; kindly provided by B. Lichty) was propagated on Vero cells (ATCC).

Murine embryonic fibroblasts (MEFs) were derived from wildtype balb-c [42], C57Bl/6(WT), TRIF^-/-^ [43], IPS-1^-/-^ [44], RIGI^-/-^ [45], MDA5^-/-^ [46] and TLR3^-/-^ [47] mice and were maintained in α-minimal essential medium (MEM) supplemented with 10% fetal bovine serum (FBS), 100 U mL^-1^ penicillin, 100 µg mL^-1^ streptomycin (pen/strep) and 2mM L-glutamine (L-glu). RAW 264.7, a murine monocyte macrophage cell line, was kindly provided by A. Ashkar. HEL, a primary human fibroblast cell line was purchased from ATCC. Both RAW 264.7 and HEL cells were grown in Dulbecco's modified Eagle's medium (DMEM) supplemented with 10% FBS, pen/strep and L-glu. Primary lung fibroblasts were derived from C57Bl/6 mouse lung explant cultures, and were maintained in DMEM medium supplemented with 20% FBS, pen/strep and L-glu. Splenocytes were identified as the adherent cells isolated from a C57Bl/6 mouse spleen that had been broken down by mechanical separation followed by ACK buffer treatment to lyse contaminating red blood cells. Splenocytes were cultured in DMEM supplemented with 20% FBS, pen/strep and L-glu, left to adhere overnight and RNA from adherent cells was isolated within 24h of culturing. All cells were incubated at 37^o^C in a humidified 5% CO_2_ incubator.

### DsRNA synthesis and treatments

DsRNA synthesis, including primer sequences, has been described previously [39]. Briefly, dsRNA was synthesized by *in vitro* transcription using the Megascript RNAi kit (Ambion). 1 µg of PCR fragments amplified from portions of the cloned West Nile virus (WNv) genome was used as a template. All three dsRNA lengths (200bp, 500 bp and 1000 bp) were based from the E protein sequence. The primers used to amplify specific genome sequences included a T7 sequence tag used by the T7 polymerase during dsRNA synthesis. The average length for the poly IC used in this study was approximately 4000 bp as determined by marker size comparison using agarose gel electrophoresis.

DsRNA treatments were performed in serum free OptiMEM media (Gibco) for specified time periods, with the first hour occurring in the presence of 50 µg/mL DEAE-dextran (Pharmacia), unless otherwise noted. DEAE-dextran is a cationic polymer that binds negatively charged nucleic acids and enables a closer association between the negatively charged cell membrane and the nucleic acid of interest [48]. In all experiments, DEAE-dextran was utilized in dsRNA-untreated controls to ensure that the polymer alone was not influencing subsequent cellular responses. DEAE-dextran was not used in the AcLDL binding assays, nor when using poly IC as a competitive ligand (Figure 1).

### Scavenger expression detection by RT PCR

Total RNA was isolated using the TRIzol reagent (Invitrogen, Burlington, ON) according to manufacturer's instruction. RNA was DNase treated using DNA-*free* as per manufacturer's instructions (Ambion, Austin, TX). cDNA synthesis was performed using 1 µg RNA, 0.2 ng of random 6mer primer, and 50 U of SuperScript II reverse transcriptase (Invitrogen). Subsequent PCR reactions were performed using 2 µL undiluted cDNA, 200nM each primer set and 1U of Taq DNA polymerase (Invitrogen) and PCR primers described in Table 1. All PCR products were sequenced to confirm identity.

### Fluorescence microscopy

DsRNA was labeled with Alexafluor 488 using the Ulysis nucleic acid labeling kit (Invitrogen). Excess labeling reagent was removed using Micro Biospin P-30 columns (BioRad, Hercules, CA). Alexafluor 488 labeled acetylated low-density lipoprotein (AcLDL) was purchased from Molecular Probes (Burlington, Canada). Cells were seeded on glass coverslips and treated with fluorescently labeled *v*200 (1 µg/mL), *v*1000 (1 µg/mL) or AcLDL (2.5 µg/mL), with or without scavenger receptor competitive ligands (all 100 µg/mL) or anti-SRA antibody and normal goat serum (ngs) control (both diluted 1∶50), as indicated. Following incubations for designated time points, cells were fixed with 4% paraformaldehyde. Nuclei were stained with Hoechst 33258 (Sigma). AcLDL binding assay were recorded as live cell images. Images were captured using a Leica DM-IRE2 inverted microscope.

### Ligand entry assay

Cells were seeded into 96 well plates and treated the next day with fluorescently labeled dsRNA or AcLDL for indicated lengths of time as described in the figure legends. Total fluorescence was measured prior to removal of unbound dsRNA. Following incubations, unbound dsRNA was removed, cells were washed with PBS, and 0.025% trypan blue was added to the wells to quench extracellular, cell-associated fluorescence to measure only intracellular fluorescence. Results were reported as a % of control cells, equalized to each respective total fluorescence.

### Endocytosis inhibitors

Cells were treated with increasing concentrations of endocytosis inhibitors chlorpromazine hydrochloride, cytochalasin D, bafilomycin A1, for 30 minutes prior to treatment with *v*1000 (1 µg/mL) in combination with the inhibitors for 2h. RNA was collected and real time PCR performed as described below. No cell death was observed for any reported concentration of inhibitor as measured using the fluorescent viability dyes, alamar Blue and CFDA-AM.

### siRNA treatment

SR-AI/II, SCARA3, SCARA4 and SCARA5 were knocked down individually or in combinations in balb-c MEFs using siRNA oligomers and reverse transfection. Gene specific oligomers (100 nM) were combined with Dharmafect1 transfection reagent and cells were seeded on top of this combination. All experiments were performed at 24h post transfection when optimal knockdown was observed. Control cultures were treated with equal amounts of a non-targeting negative control pool of oligomers.

### Real time RT- PCR

For real time RT-PCR, cells were treated with dsRNA in the presence or absence of endocytosis inhibitors, scavenger receptor ligands, antibodies or siRNA oligomers. After specified time points, RNA was isolated from treated MEFs using Trizol reagent (Invitrogen) according to the manufacturer's protocol. RNA was DNase treated using DNA-*free* as per the manufacturer's specifications (Ambion), and then quantified using the Agilent 2100 Bio-Analyzer (Agilent, Santa Clara, CA). 200 ng of total RNA was reverse transcribed with 0.2 ng of random 6mer primer and 50 U of Superscript II (Invitrogen) in a total reaction volume of 20 µL. Real-time quantitative PCR was performed in triplicate, in a total volume of 25 µL, using Universal PCR Master Mix and gene specific primers (Applied Biosystems). PCR was run in the ABI PRISM 7900HT Sequence Detection System using the Sequence Detector Software version 2.2 (Applied Biosystems). Data were analyzed using the ΔΔC_t_ method. Specifically, gene expression was normalized to the housekeeping gene (GAPDH) and expressed as fold change over the control group.

### Animal experiments

SR-AI/II^-/-^ mice and wild type (WT) C57Bl/6 control mice were used for these experiments. The age and sex-matched control mice were purchased from Charles River Laboratories (Montreal, PQ, Canada). Animals were anesthetized and 50 µg poly IC was delivered intranasally in 35 µl of phosphate-buffered saline (PBS) vehicle. Mice were sacrificed 12 hours following treatment with poly IC. Lungs were removed, tracheas cannulated (Becton Dickinson and Co., Sparks, Maryland, USA) and broncho-alveolar lavage fluid (BALF) obtained by lavaging twice with PBS (250 µl and 200 µl). BALF was stored on ice until antiviral assays were performed as described below. Three lobes from the multi-lobed side of the lung were minced and stored in RNAlater (Ambion, Streetsville, Canada) at −80°C until RNA isolation and real time PCR was performed as described above.

### Antiviral assay

For BALF the antiviral assay was performed as previously described [49]. Briefly, cells were removed from BALF samples by centrifugation and monolayers of MEFs were incubated with serially diluted supernatants for 24 hours in 24 well dishes. To measure the dsRNA induced antiviral response, MEFs (WT, TLR3^-/-^, TRIF^-/-^, RIGI^-/-^, MDA5^-/-^ IPS-1^-/-^) were treated with serial dilutions of equal nanomolar amounts of poly IC or *in vitro* transcribed dsRNA (*v*200) for 6h. In all cases supernatants were removed and MEFs were infected with VSV-GFP (MOI of 0.1) in serum free α-MEM for 1h. Viral inoculate were replaced with DMEM containing 1% methylcellulose and GFP fluorescence intensity was measured 24 hours later on a Typhoon Trio (GE Healthcare) and quantified using the ImageQuant™TL software. For dsRNA responses, a dose response curve was generated for each cell line.

### Western blot analysis

For Western blot analysis, cells were washed twice in ice-cold PBS, and collected by centrifugation at 200 xg for 4 min at 4°C. Cell pellets were lysed in a non-reducing whole cell extract buffer (20 nM HEPES [pH 7.4], 100 mM NaCl, 10 mM β-glycerophosphate, 0.2% Triton X-100, 50 nM sodium fluoride, 1mM sodium orthovanadate, 1mM phenylmethylsulfonyl fluoride, and 1X protease inhibitor cocktail [Sigma]), for 15 min on ice, and then cleared by centrifugation for 10 min at 13,000 xg at 4°C. 20 µg of cell extract was run on 10% polyacrylamide gels, transferred onto nitrocellulose membrane, and probed with anti-human SR-A (1∶2000). Blots were then incubated with anti-goat horseradish peroxidase -conjugated secondary antibody and visualized using an enhanced chemiluminescence system (ECLplus kit, GE Healthcare).

### Statistical analysis

Data are expressed as means ± standard error of the mean (unless otherwise indicated). Statistical analysis was performed using a one-way analysis of variance (ANOVA) with a tukey's *post hoc* test for pair-wise comparisons, a Dunnett's post test for comparisons with control treatments, and an unpaired t-test in the case of a comparison between two values. All statistical analysis was performed using GraphPad InStat (version 3.00 for Windows 95, GraphPad Software, San Diego California USA, www.graphpad.com). A *p* value of <0.05 was considered statistically significant.

## Supporting Information

Figure S1 MEFs derived from balb-c mice bind dsRNA similarly to C57Bl/6 MEFs. Balb-c derived MEFs were treated for 1h with 1 µg/mL Alexfluor 488 labeled *v*200 or *v*1000 in the presence or absence of fucoidin or fetuin (both 100 µg/mL). Cells were fixed and nuclei were counterstained with Hoescht 33258. Magnification 400X.
(0.72 MB TIF)
