# Supplementary figures and images for: An Accessory to the ‘Trinity’: SR-As Are Essential Pathogen Sensors of Extracellular dsRNA, Mediating Entry and Leading to Subsequent Type I IFN Responses
Source: PLoS Pathog. 2010 Mar 26;6(3):e1000829. doi: 10.1371/journal.ppat.1000829 (PMC2847946; doi:10.1371/journal.ppat.1000829)

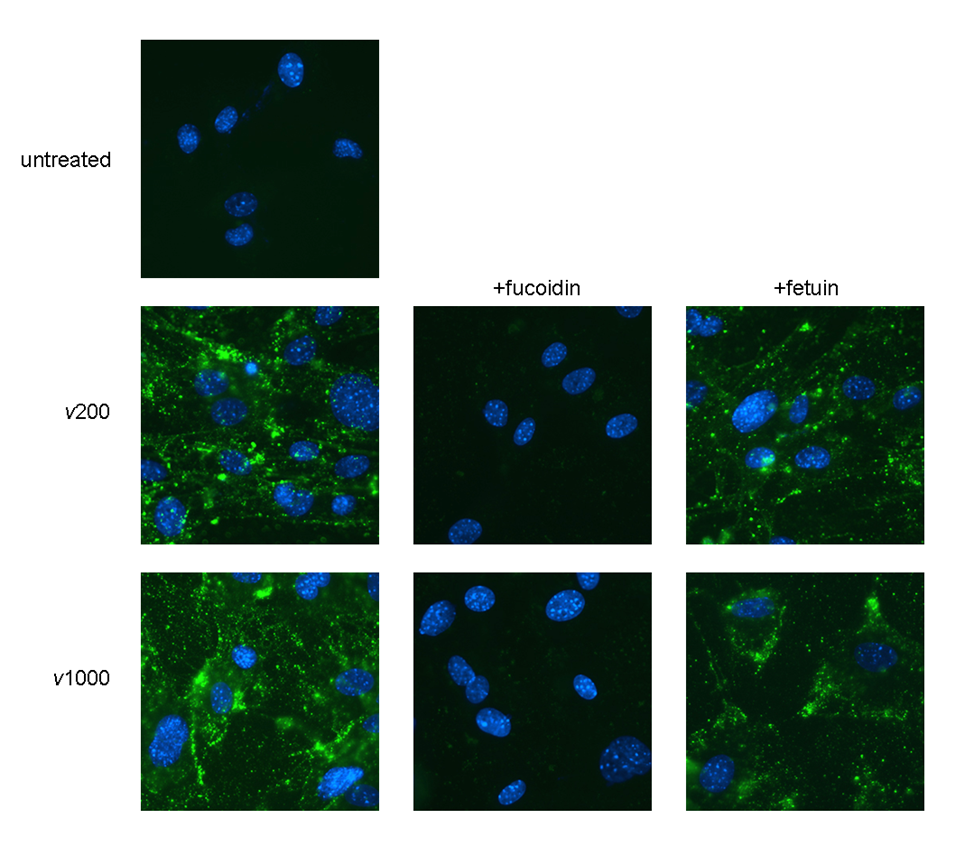

Supplement: Figure S1 — MEFs derived from balb-c mice bind dsRNA similarly to C57Bl/6 MEFs. Balb-c derived MEFs were treated for 1h with 1 µg/mL Alexfluor 488 labeled v200 or v1000 in the presence or absence of fucoidin or fetuin (both 100 µg/mL). Cells were fixed and nuclei were counterstained with Hoescht 33258. Magnification 400X. (0.72 MB TIF) [file ppat.1000829.s001.tif]
